# Supplementary material for: Effect of Phosphorylation on the Collision Cross Sections of Peptide Ions in Ion Mobility Spectrometry
Source: Mass Spectrom (Tokyo). 2021 Jan 30;10:A0093. doi: 10.5702/massspectrometry.A0093 (PMC7843839; doi:10.5702/massspectrometry.A0093)
Supplement: Supplementary Data [file massspectrometry-10-1-A0093_s001.pdf]

SUPPORTING INFORMATION

Effect of Phosphorylation on the Collision Cross Sections of Peptide Ions in Ion Mobility Spectrometry

Kosuke Ogata[1], Chih-Hsiang Chang[1] and Yasushi Ishihama[1*]

1 - Department of Molecular and Cellular BioAnalysis, Graduate School of Pharmaceutical Sciences, Kyoto University, Sakyo-ku, Kyoto, Japan, 606-8501

*Correspondence and requests for materials should be addressed to Y.I. (email: yishiham@pharm.kyoto-u.ac.jp).

Table of Contents

Supplementary Figure S1. Visual classification of extended and compact forms of triply charged unphosphopeptides.

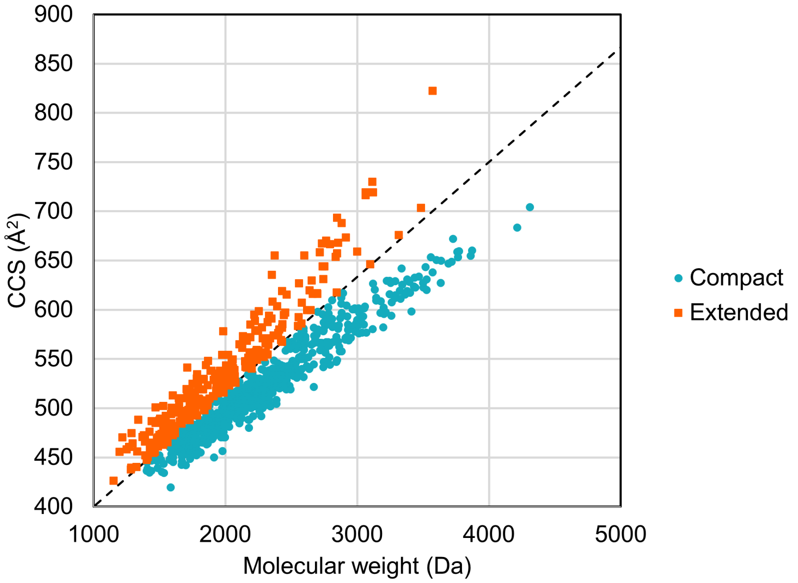

Supplementary Figure S1.

Visual classification of extended and compact forms of triply charged unphosphopeptides. The dashed line represents the classification boundary.
